# Phylogeography of screaming hairy armadillo *Chaetophractus vellerosus*: Successive disjunctions and extinctions due to cyclical climatic changes in southern South America

**DOI:** 10.1371/journal.pone.0190944

**Published:** 2018-01-11

**Authors:** Sebastián Poljak, Alejandro M. Ferreiro, Marina B. Chiappero, Julieta Sánchez, Magalí Gabrielli, Marta S. Lizarralde

**Affiliations:** 1 Laboratorio de Ecología Molecular, Centro Austral de Investigaciones Científicas, Consejo Nacional de Investigaciones Científicas y Tecnológicas, Ushuaia, Tierra del Fuego, Argentina; 2 Instituto de Ciencias Polares, Ambiente y Recursos Naturales, Instituto de Ciencias Polares Ambiente y Recursos Naturales, Universidad Nacional de Tierra del Fuego, Ushuaia, Tierra del Fuego, Argentina; 3 Cátedra de Genética de Poblaciones y Evolución, Facultad de Ciencias Exactas, Físicas y Naturales, Universidad Nacional de Córdoba, Córdoba, Argentina; 4 Consejo Nacional de Investigaciones Científicas y Técnicas, Instituto de Diversidad y Ecología Animal, Córdoba, Argentina; 5 Laboratorio de Ecología Molecular, Centro Regional de Estudios Genómicos, Facultad de Ciencias Exactas, Universidad Nacional de La Plata, La Plata, Buenos Aires, Argentina; National Cheng Kung University, TAIWAN

## Abstract

Little is known about phylogeography of armadillo species native to southern South America. In this study we describe the phylogeography of the screaming hairy armadillo *Chaetophractus vellerosus*, discuss previous hypothesis about the origin of its disjunct distribution and propose an alternative one, based on novel information on genetic variability. Variation of partial sequences of mitochondrial DNA Control Region (CR) from 73 individuals from 23 localities were analyzed to carry out a phylogeographic analysis using neutrality tests, mismatch distribution, median-joining (MJ) network and paleontological records. We found 17 polymorphic sites resulting in 15 haplotypes. Two new geographic records that expand known distribution of the species are presented; one of them links the distributions of recently synonimized species *C*. *nationi* and *C*. *vellerosus*. Screaming hairy armadillo phylogeographic pattern can be addressed as category V of Avise: common widespread linages plus closely related lineages confined to one or a few nearby locales each. The older linages are distributed in the north-central area of the species distribution range in Argentina (i.e. ancestral area of distribution). *C*. *vellerosus* seems to be a low vagility species that expanded, and probably is expanding, its distribution range while presents signs of genetic structuring in central areas. To explain the disjunct distribution, a hypothesis of extinction of the species in intermediate areas due to quaternary climatic shift to more humid conditions was proposed. We offer an alternative explanation: long distance colonization, based on null genetic variability, paleontological record and evidence of alternance of cold/arid and temperate/humid climatic periods during the last million years in southern South America.

## Introduction

The evolution of the Order Xenarthra was bound to South America as they have been considered to be representatives of the initial mammalian stock in this continent [1]. It is the least diversified group of mammals but, as old South American endemics, they constitute a good model for understanding the biogeographical and diversification patterns present in South America [2]. Armadillos (grouped in the families Dasypodidae and Chlamyphoridae) are the most diverse xenarthran lineage. Phylogeographic and phylogenetic studies as those of Delsuc et al. [3, 4], Poljak et al. [5], Loughry & McDonoug [6], Abba et al. [7], Moraes Barros et al. [8] among others, provided information about South American biogeographic history and general patterns of species diversification in this group.

The screaming hairy armadillo *Chaetophractus vellerosus*, or pichi llorón, presents fossorial habits, an omnivorous diet and is nocturnal during summer but diurnal during winter when individuals leave their burrows around noon, at the warmest few hours of the day [9, 10]. It is one of the most widely distributed armadillo species: its range follows approximately the arid and semiarid regions of the center and northwest of Argentina, southeastern Bolivia and northeastern Paraguay [10]. A recent work shows that *Chaetophractus nationi* and *C*. *vellerosus* would be the same species, and thus its distribution would extend further to north in Bolivia [7]. The species prefers areas with loose, sandy-calcareous soils and is well adapted to arid and semiarid conditions. This adaption to xeric environments is reflected by some physiological and behavioral characteristics like the capacity of individuals to maintain their water balance in dehydration conditions, similar kidney functions to those found in other aridity adapted mammals [11] and a relatively high basal metabolism rate [12].

**Fig 1:**
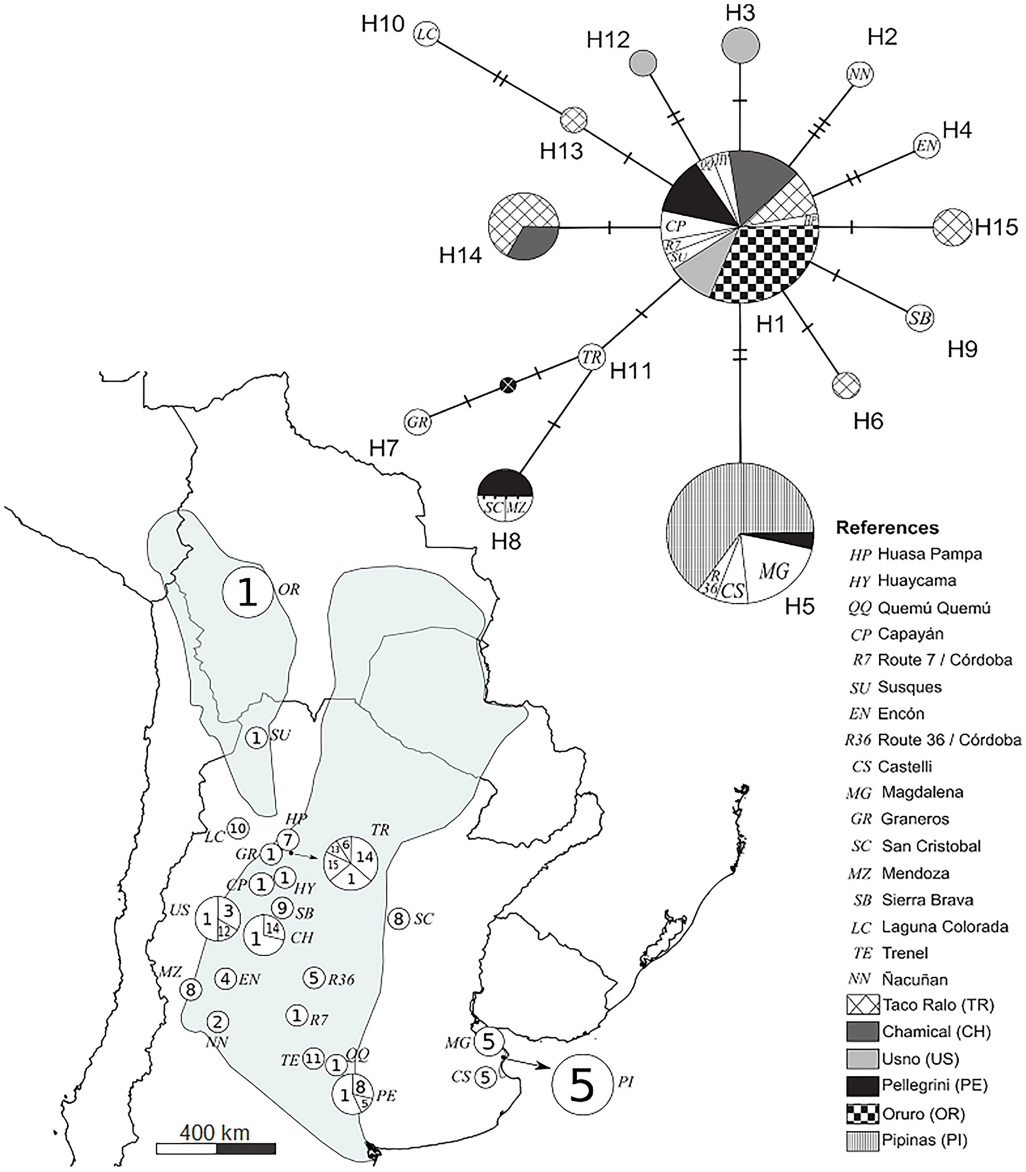
Network linking *C*. *vellerosus* CR haplotypes (H1, H2…Hn). Cross hatches on the net represent nucleotide differences between haplotypes. Circle sizes depend on haplotypes relative frequencies. Localities of origin of the samples are listed in “References”. Their geographic locations are depicted on the map with circles which have the same reference code. Size of these circles depends on number of individuals sampled in the locality and numbers in circle portions represent the relative frequencies of haplotypes found in each locality.

The currently distribution range of *C*. *vellerosus* in Argentina encompass the provinces of Jujuy, Salta, Formosa, Chaco, Catamarca, Tucumán, Santiago del Estero, San Juan, La Rioja, Mendoza, San Luis, Córdoba, La Pampa and Buenos Aires [13]. Remarkably, the species has a disjunct population over the Atlantic coast of Samborombón Bay in Buenos Aires province, 500 km to the east from the core distribution [14] (Fig 1). To explain this current disjunction, the authors proposed that the species extended its distribution range toward the Atlantic coast during a pulse of aridity. Then, due to a climatic shift to humid conditions at the end of the Pleistocene and part of the Holocene, the coastal population became disjunct by extinction of the intermediate populations.

Subsequent studies made by Rabassa et al. [15] determined that cold and dry glacial periods alternated with warm and humid interglacial ones 14 to 16 times, affecting especially the Andes Mountains and central and southern regions of the Southern Cone. These changes determined large sea level variations that repeatedly modified the configuration of emerged lands [16], and particularly in the Pampean Region produced the alternation of savannahs and arid steppes with more humid tropical and subtropical forests [17]. Edaphic characteristics of Bahía Samborombón (well-drained sandy sediments), may have favored the permanence of the species and led to the disjunct distribution observed nowadays even though actual more humid interglacial conditions.

On the other hand, Soibelzon et al. [18] assigned fossil remains from a coastal locality of Buenos Aires province called Punta Hermengo to *C*. *vellerosus* (see Fig 1) and estimated that their age could be 0,8 My. This finding suggests that the current disjunct coastal population can have a history as long as the estimated fossil age.

In this work we aim to contribute to the knowledge of the biogeographic history of South American xenarthrans by describing the phylogeographic patterns of *C*. *vellerosus* populations using new molecular data combined with fossil record and contrasting previous hypothesis based on biogeography and past climate changes to explain the current disjunct distribution of this species.

## Results

### Specimens analysed, sample locations and sequence variation

We obtained CR fragment sequences 456 bp long from 73 individuals of 23 localities (in S1 Table and Fig 1). Two of these localities: Laguna Colorada (Catamarca province) and San Cristóbal (Santa Fe province), are new records for the distribution of the species. It was not possible to obtain the DNA sequences from the rest of the samples probably due to the poor preservation of the tissues and the chemical treatments of the skins of the museum specimens, among other possible causes.

Aligned sequences showed 17 polymorphic sites (12 transitions and 5 transversions) resulting in 15 haplotypes. The nucleotide composition of sequences was 26.91% C, 29.39% T, 31.06% A, 12.64% G and mean nucleotide diversity (π) among all haplotypes was 0.006460 +/- 0.003750. Samples from Bolivia and from the disjunct populations were monomorphic for haplotypes H1 and H5 respectively.

Haplotypes, polymorphic sites, relative frequencies and GenBank accession numbers are given in Table 1. Distribution of the species including new localities and haplotypes in each location are given in Fig 1.

**Table 1:** Haplotypes of *C*. *vellerosus* Control Region partial sequences and its polymorphic sites. Haplotypes, polymorphic sites, relative frequencies (Rel.freq), GenBank name (Gbank name) and accession numbers (Gbank acc.) of *C*. *vellerosus* sequences.

Haplotype	Polimorphic sites																	Rel.freq.	Gbank name	Gbank acc.
	12	77	114	155	189	230	235	248	249	260	289	316	321	348	356	380	424			
H1	A	C	T	A	A	C	A	G	T	C	C	A	T	A	C	A	T	0.39506	CRvelle1	DQ136318
H2	G	T												G				0.01234	CRvelle2	EU090356
H3			C															0.02469	CRvelle3	EU090357
H4				T			T											0.01234	CRvelle4	EU090358
H5					G					T								0.32098	CRvelle5	EU090359
H6						T												0.01234	CRvelle6	FJ824592
H7						T					T				T			0.01234	CRvelle7	FJ824594
H8						T		A			T							0.04938	CRvelle8	FJ824595
H9								A										0.01234	CRvelle9	FJ824596
H10									C				G	G				0.01234	CRvelle10	MG020351
H11											T							0.01234	CRvelle11	MG020352
H12												T				C		0.01234	CRvelle12	MG020353
H13													G					0.01234	CRvelle13	MG020354
H14															T			0.07407	CRvelle14	MG020355
H15																	C	0.02469	CRvelle15	MG020356

#### Phylogeographic analysis

Fig 1 illustrates the network linking *C*. *vellerosus* CR haplotypes and their source localities. H1 can be considered the oldest (ancestral) haplotype because it occupies a central position in the network. It is present in 11 of the 23 sampled sites, latitudinally located along the entire distribution range of the species and other 11 haplotypes derive from it. Locations with the highest number of haplotypes were Taco Ralo (TR) = 5 (H1, H6, H13, H14, y H15), Usno (US) = 3 (H1, H3, H12), Pellegrini (PE) = 3 (H1, H5, H8) and Chamical (CH) = 2 (H1, H14). In the remaining sampling sites, only one haplotype was registered although the number of sampled individuals was high, as in the case of Pipinas (n = 17). No variability was found among Bolivian samples, H1 was the only haplotype present (see S2 Table for details).

**Fig 2:**
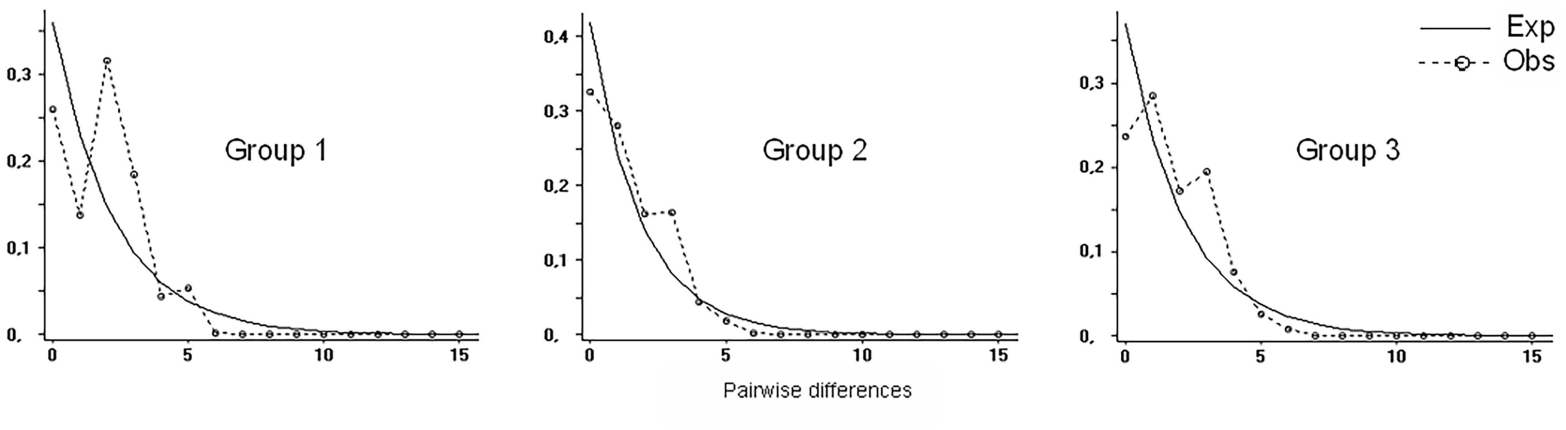
Compared mismatch distributions of the three groups of samples. Group 1 includes all samples, Group 2 excludes samples from the coastal disjunct population and Group 3 excludes samples from the coastal disjunct population and from Bolivia). Exp: expected, Obs: observed. Mean number of pairwise differences, sum of square deviations, Harpending´s raggedness index, Tajima´s D and Fu´s index for each group of samples are presented in Table 2.

Mismatch distributions of different groups are presented in Fig 2. Observed mismatch values do not differ significantly from expected values in the three groups of samples. However, Group 1 shows a greater difference between observed and expected values, because the disjunct coastal population and the Bolivian samples are monomorphic for haplotypes H5 and H1 respectively. These haplotypes differ in two mutational steps, and their high sample size affects the modes at 0 and 2.

Considering the genealogy of haplotypes and its geographic distribution, *C*. *vellerosus* would have experienced different processes. In the case of *contiguous range expansion* [19, 20], some ancestral haplotypes are distributed in the pre-expansive area of distribution, while younger haplotypes arise in population expansion areas and are geographically dispersed (*contiguous range expansion*) or located in distant areas from those occupied by ancestral ones (*long distance colonization*). In *restricted gene flow with isolation by distance* pattern [21], the distribution of a derived haplotype is geographically restricted, and coincides with the distribution of its ancestral haplotype. Patterns of linages distribution associated with restricted gene flow have been investigated using computer simulations by Neigel et al. [22], Neigel and Avise [21], Nath and Griffiths [23] and Slatkin [24, 25]. Theoretical results show that geographical extent of haplotypes is strongly correlated with their age (i.e, the older the haplotype, the more widespread it tends to be under a restricted gene flow model). The positive relation between restricted gene flow, haplotype age and distribution could be the cause of the isolation by distance pattern typical of species with low vagility [26, 27]. This is not a historical process, but leaves footprints in genetic population structure because it occurs generation after generation.

**Fig 3:**
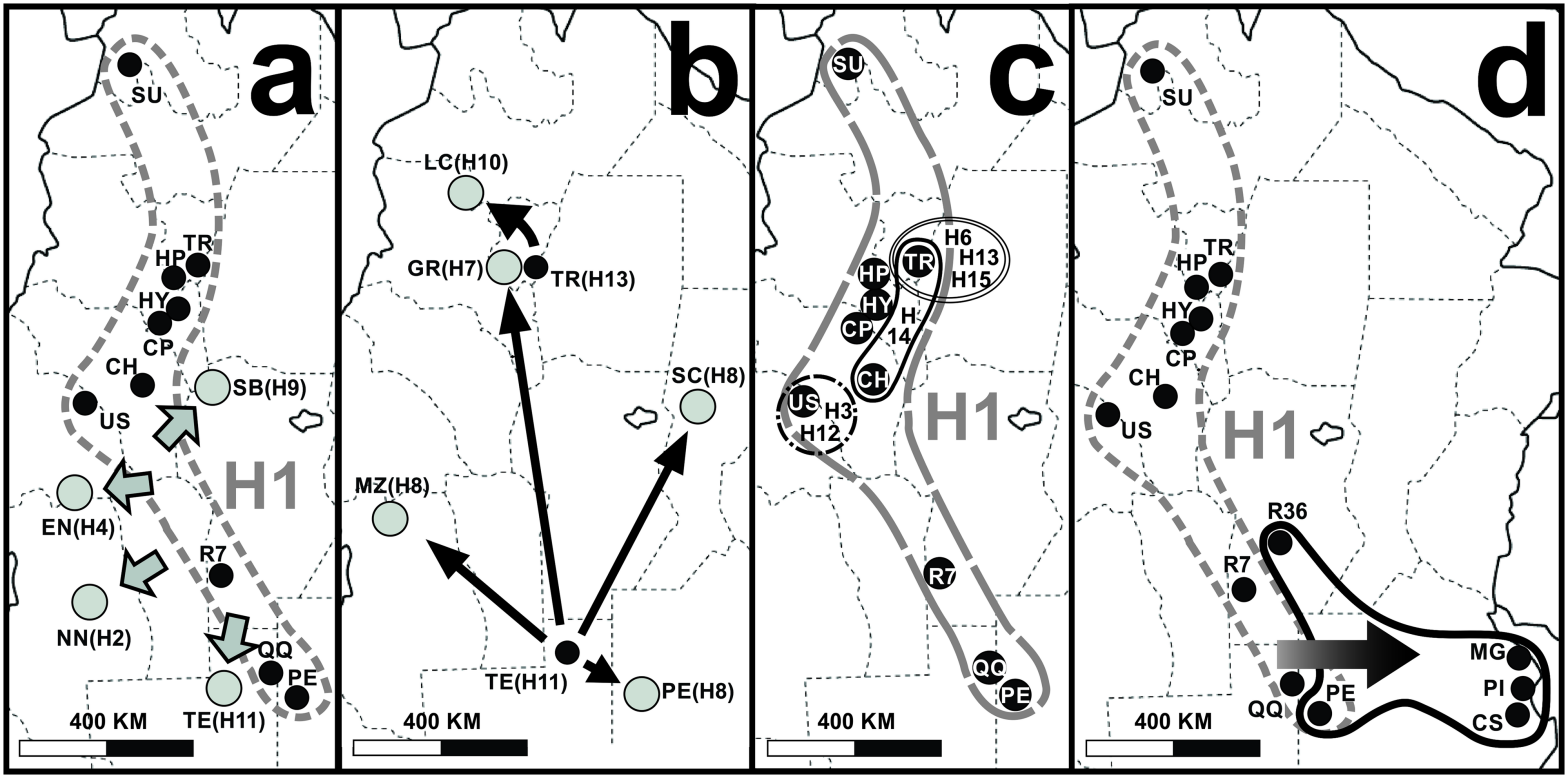
*C*. *vellerosus* phylogeographic patterns depicted separately. 3a) and 3b) *Contiguous range expansion*; 3c) *Restricted gene flow with isolation by distance*; 3d) *Long distance colonization*. Black and grey dots: localities (codes following Fig 1 and S1 Table); haplotypes are between brackets; arrows indicate the direction of geographic expansion of derived haplotypes from their ancestor (H1 in 3a and 3d; H11 and H13 in 3b); grey segmented lines: distribution of H1 haplotype; black thin segmented line: geographic distribution of H3 and H12 haplotypes; black thin solid line: geographic distribution of H14; double black thin solid line: geographic distribution of H6, H13 and H15 haplotypes; black thick solid line: distribution of H5 haplotype.

These patterns were observed in the phylogeography of *C*. *vellerosus* and can be visualized in the following relationships between haplotype genealogies and geographical distribution (Hn: haplotypes; codes of localities in capital letters) extracted from Fig 3.

1. *Contiguous range expansion* (Fig 3a and 3b):
  - Haplotype H1 is distributed in HP, TR, CH, HY, QQ, PE, CP, R7, SU, US and OR. Its derived haplotypes H2, H4, H9 and H11 are distributed in TE, NN, EN and SB respectively.
  - Haplotype H13 is distributed in TR. Its derived haplotype H10 is distributed in LC.
  - Haplotype H11 is distributed in TE. Its derived haplotype H7 is distributed in GR and H8 is distributed in MZ, SC and PE.
2. *Restricted gene flow with isolation by distance* (Fig 3c):
  Haplotype H1 is distributed in HP, TR, CH, HY, QQ, PE, CP, R7, SU, US and OR. Its derived haplotypes H3 and H12 are distributed in US; haplotypes H6, H13, H15 are distributed in TR and haplotype H14 is distributed in TR and CH.
3. *Long distance colonization* (Fig 3d):
  Haplotype H1 is distributed in HP, TR, CH, HY, QQ, PE, CP, R7, SU, US and OR. Its derived haplotype H5 is distributed in R36, PE, PI, MG and CS.

## Discussion

### Sequence variation

The nucleotide composition of *C*. *vellerosus* sequences is similar to values previously reported for related species as *Chaetophractus villosus* [5]. However, mean nucleotide diversity (π) among all haplotypes is higher in *C*. *vellerosus* than in *C*. *villosus* (0.007679 +/- 0.004389 and 0.003781 +/- 0.002436 respectively).

### Neutrality tests and mismatch distributions

Neutrality tests reject the null hypothesis of population stability (D or Fs = 0) for the three sample sets (Table 2). In *Data sets 1* and *2*, negative values of Tajima’s D indicate population expansion, while a significant positive value was obtained for *Data set 3*. A positive Tajima’s D signifies low levels of low and high frequency polymorphisms, indicating a decrease in population size (which may be due to a recent bottleneck) and/or balancing selection. On the other hand, Fu’s tests results are negative and significant for all sample sets. Fs takes negative values when an excess of rare haplotypes are found, phenomenon that occurs when populations experienced a recent expansion. Harpending´s raggedness index also supports a population expansion for this species for the three sample sets. These results indicate that *C*. *vellerosus* populations would have experienced an expansion. However, we wish to highlight some features of *Data set 3* in order to give relevance to positive Tajima´s test results that in principle seem contradictory but could be reflecting an ongoing process toward genetic population stability disguised by recent expansions of H1 and H5 linages. We have to consider that: 1) all haplotypes were found in *Data set 3*, and relative frequencies do not show as large differences between populations as in *Data set 1* and in *Data set 2*; 2) *Data set 3* includes the central haplotype H1 ancestral to all others and other haplotypes located in intermediate positions in the genealogy as H11 and H13, relationships that need time to develop; 3) This genealogy suggest that the area where old haplotypes are distributed can be considered an ancestral distribution range of the species, and 4) the mean number of pairwise differences of *Data set* 3 are higher than in other groups, reflecting a higher genetic variability (Table 2). These points would indicate that *C*. *vellerosus* populations are indeed expanding in the marginal areas of its range distribution, but central populations would be more stable and would show an incipient genetic structuring.

**Table 2:** Demography of *C*. *vellerosus* population. Number of samples (n), mean number of pairwise differences (Mean n° of pairwise differences), sum of square deviations (SSd), Harpending´s raggedness index, Tajima´s D and Fu´s (Fs) index for each group of samples.

Groups	n	Mean n° of pairwise differences	SSd	Harpending’s raggedness index	Tajima’s D	Fs
1	81	3.133 +/- 1.641	0.0244 P: 0.214	0.0813 P: 0.316	-0.246 P ˂ 0.001	-2.320 P ˂ 0.001
2	57	3.308 +/- 1.725	0.0129 P: 0.544	0.0481 P: 0.838	-0.313 P ˂ 0.001	-3.023 P ˂ 0.001
3	47	3.987 +/- 2.030	0.2420 P: 0.002	0.0361 P: 1.000	0.113 P ˂ 0.001	-2.618 P ˂ 0.001

### Phylogeography

*C*. *vellerosus* phylogeography cannot be easily classified into a specific phylogeographic category, but shows patterns similar to those that define category V described by Avise [26]: species with common linages that are widespread, plus closely related lineages each confined to one or a few nearby locales. In particular, the presence of a single haplotype H1 in all Bolivian localities included in this study (and SU in north-west Argentina, Fig 1, S1 and S2 Tables) and the analogous situation of H5 in the Eastern part of the Pampean Region towards the Atlantic coast, could be explained by a distribution range expansion with drastic loss of genetic variability. A loss of genetic variability in the advance front of colonization has been described in other armadillo species such as nine-banded armadillos in North America, where individuals share a single divergent monomorphic haplotype, even for those separated by 1000 km, possibly fixed after severe drift [28]. A similar situation was observed in *C*. *villosus* colonization front to Argentinian Patagonia, where a single haplotype is distributed along 1500 km to the south [5]. However, unlike nine banded armadillo, this single *C*. *villosus* haplotype is also present in other northern localities as is the case of haplotype H5 of *C*. *vellerosus*, present in both the disjunct coastal population and the core distribution.

In the northwestern expansion front H1 is the only haplotype found, although the twelve sequences included in this study belong to individuals from Bolivian localities spread in an area about 27.000 km² (geographic location in S2 Table). The same haplotype was found in SU (Argentina), more than 600 km far to the south. Taking into account that the distribution of *C*.*vellerosus* to the northwest is continuous [7], this seems to be a case of contiguous range expansion with loss of genetic variability. However, by definition in *contiguous range expansion* some ancestral haplotypes are distributed in the pre-expansive area of distribution, while younger haplotypes arise in population expansion areas and are geographically dispersed [20]. Therefore, the expansion of *C*. *vellerosus* to the northwest does not fit the definition since H1 is the ancestral haplotype and is distributed in all the core distribution of the species. Further samplings in intermediate areas are needed to know the rate of loss of variability in relation to the advance of colonization wave.

Related to the expansion to the east, disjunct distribution of *C*. *vellerosus* was previously explained by means of an expansion (i.e. *contiguous range expansion*) followed by extinction of intermediate populations. Poljak et al. [5] described two distribution range expansions of the sister species *C*. *villosus* to the east of Pampean Region. These expansions comprised two lineages that split up in different times, probably related to glaciations, without a doubt the most important events that affected climatic and environmental conditions in southern South America in the past. *C*. *villosus* (as well as other related armadillo species) are recorded in several paleontological and archeological sites in the east of Pampean Region (i.e. the intermediate area of *C*. *vellerosus*). If we accept the hypothesis that *C*. *vellerosus* expanded its distribution and reached the coast 0.78 Mya during middle Pleistocene, then became disjunct and after that experienced secondary contacts with the core distribution due to glacial periods of arid climate, it is difficult to explain its current lack of genetic variability. The only haplotype present in the disjunct population (H5) presents the second wider distribution range between all *C*. *vellerosus* haplotypes found in this study after H1 and followed by H8 (see Fig 1). A wide distribution needs time to develop; specially taking into account that *C*. *vellerosus* is a small mammal, with small home ranges [29] and low vagility. Therefore, these haplotypes could be considered older than others and indicates that H5 is not a young haplotype that appeared in a new area of colonization. Besides, frequency of H1 and H8 are higher than H5 in PE, the most border locality of the species core distribution to the east (Fig 1) and hence the closest to the disjunct population. If we consider the hypothesis that there have been secondary contacts between core and disjunct populations, and given that H5 shares similar characteristics with H1 and H8 (distribution pattern, old haplotypes, present in PE), why H5 is the only haplotype present in the disjunct population? Genetic drift could be an explanation; it could have reduced the variability, keeping it null after the last wave of colonization until today. But, for this to have happened, the population must have been small during at least 15000–10000 years, the age of the last glaciation [15]. Furthermore, we have to consider that the current distribution of the disjunct population encompass localities separated for more than 100 km and covers an area about 4500 km². Due to the strong association of *C*. *vellerosus* with loose sandy soils [9, 10], its absence in low lands [30] and ecological features mentioned before, the species presents a patchy distribution. Under these conditions, genetic drift in combination with gene flow from the core area should have led to an increase in variability among patches within the disjunct population. By contrast, in *long distance colonization* usually ancestral haplotypes are distributed in the pre-expansive area of distribution, while younger haplotypes are located in distant areas from those occupied by ancestral ones. *C*. *vellerosus* expansion to the east does not fit exactly with this definition since H5 is a derived-from-H1 haplotype that occupies a distant area from core distribution but it is also found in some populations within it. However, its presence in the disjunct area could be explained by translocation to the coast by the human activity from this area that may have occurred in very recent times. This would also explain the null variability present in the area. Some paleobiogeographic considerations discussed below may help to clarify this issue.

#### Palaeobiogeographic considerations

Every taxon inhabits certain areas with characteristic variables; if they change, each taxon has two different outcomes: local extinction or displacement to an area where the variables have not changed [31]. Among the factors that could have influenced historic climatic changes in the Pampean Region, Patagonic glaciations and the concomitant changes in sea level were among the most influential in the Pampean Region. *C*. *vellerosus* is an arid-adapted species, so it is reasonably to suppose that colonization towards east coast would have been possible due to cyclic aridity pulses associated with the advance of glaciers and with the increase of the exposed continental surface due to the decrease of sea levels. The global oceanic oxygen isotopic sequence, magnetostratigraphic records and glaciations records (see [15, 32, 33]) can perfectly explain cyclic periods of good (arid) conditions that would have allowed *C*. *vellerosus* to colonize the disjunct area that currently occupies. Three of this arid periods were especially strong and drastically affected the biotas [34].

The Quaternary record of Euphractinae and Tolypeutinae shows displacements (expansions/contractions) to eastern Pampean Region that surely occured more than one time during glacial/interglacial cycles, probably in coincidence with glaciations and/or arid phases of the Holocene ([35] and references therein). Also Tonni [36] and Tonni et al. [37] stated that the conditions during almost all the Holocene were of aridity/semiaridity and would have allowed the expansion of Patagonian and central Argentinean species as it had occurred previously during the Pleistocene. This event probably favored the expansion of *T*. *matacus* and *Z*. *pichy* into the Pampean Region during the Middle Holocene [38]. However, in the literature regarding fauna associations from Pleistocene-Holocene and archaeological sites of Buenos Aires Province there are no records of *C*. *vellerosus* in the intermediate zone between its core distribution and the coastal disjunct population, despite other closely related armadillo species like *C*. *villosus*, *Z*. *pichiy* and *T*. *matacus* are indeed present ([39–42] among others). An insightfull study of the fauna associations in 10 fossil localities in central-southern Buenos Aires province, comprising from the early Miocene to recent, doesn’t mention the presence of *C*. *vellerosus* [39]. However the species is currently present in the area, around the city of Bahía Blanca ([43]; see also IUCN map http://maps.iucnredlist.org/map.html?id=89604632). In a recent study of fauna association in an archeological site of a coastal locality in southwest Buenos Aires, 40 km to south from Punta Hermengo, Soibelzon & León [38] reported the presence of *Z*. *pichiy* and *T*. *matacus* of about 5000 years old, but does not mention *C*. *vellerosus*. This finding delimits the age of the last arrive of this species to the area. The lack of genetic variability of disjunct population is in agreement with a recent re-colonization of the area (possibly less than 5000 years).

Three alternative hypotheses can be proposed regarding the re-colonization routes that *C*. *vellerosus* may have followed toward the coast. The first hypothesis is based on [44], that reports the presence of *Tolypeutes matacus* in the north of Buenos Aires province in the Pleistocene-Holocene limit, and postulates that its range expansion was possible due an aridity pulse. This species is also present in several faunal associations throughout the Pleistocene in the Atlantic coast and nearby localities [40, 45]. The current distribution of *C*. *vellerosus* overlaps most of the distribution of *T*. *matacus* (see http://maps.iucnredlist.org/) so probably both species would share similar environmental requirements. Therefore, it is plausible that *C*. *vellerosus* may have reached the coast by the north of Buenos Aires province. An alternative colonization route from south along the Atlantic coast of Buenos Aires province can be proposed based on the work of Soibelzon et al. [18] and Soibelzon & Leon ([38] and references therein). In the first work they describe paleozoogeographical and paleoclimatic aspects related to the fossil of *C*. *vellerosus* from the coastal locality Punta Hermengo, who lived there 0.78 million years ago. In the second, they argue that expansion of *C*. *vellerosus* and *T*. *matacus* from central Argentina to the southeast Pampean Region during glacial/interglacial cycles was probably across the "Argentinean arid diagonal" (or "South American transition zone” sensu Morrone [46]). Given that *C*. *vellerosus* is currently present in southwest Buenos Aires province (Fig 1), it is possible to assume that it could have reached its current location in the coast from south. This “south route hypothesis” seems stronger that the north one, but further samplings all along the east marginal populations of the core distribution are needed to solve this issue. A third hypothesis can be proposed, based on Abba et al. [30]. These authors analyzed characteristics of *C*. *vellerosus* individuals from the disjunct population (who live in humid conditions) and of northwestern individuals (living in aridity). Based on the premise that the disjunct population is a relict of an old, wider past distribution, they expected to find different ecological characteristics between individuals of both populations (like adaptation to more arid conditions in western individuals, and to humid conditions in eastern individuals). However, they found no differences between them. A possible explanation would be a modern translocation to the bonaerian coast of a few individuals by humans, given that it was demonstrated that *C*. *vellerosus* was part of the medium sized species consumed by the hunter-gatherers since the Holocene in different archaeological sites in Córdoba [47], Santiago del Estero [48–50] and possibly in Buenos Aires [51].

For the reasons set above, we consider that the hypothesis of isolation due to the climatic changes during the Quaternary that produced the extinction of the species in the intermediate area and resulted in the disjunct distribution is not supported. Alternatively, considering that: 1) *C*. *vellerosus* inhabited the coast 0.78 Mya, 2) currently there is no genetic variability in an hypervariable fragment of mtDNA in coastal populations, 3) the only haplotype present in the disjunt population is an ancestral haplotype, present in the eastern border of the core population and 4) the alternation of humid warm/dry arid conditions in the area occurred 14 to 16 times during the last million years [15], we propose a long distance colonization as alternative hypothesis to explain the presence of the disjunct population of *C*. *vellerosus* in the Atlantic coast of Buenos Aires province, possibly caused by translocation of individuals by human activity.

## Conclusions

*C*. *vellerosus* population is expanding its distribution range and the front waves of colonization suffer a strong loss of genetic variability, as was reported for other armadillo species. The species reach the Atlantic coast at least twice, one 0.78 million years ago and the other recently. Based on cyclical climatic changes, the evidence of biogeographic response of closely related armadillo species and paleontological and archaeological records in the intermediate area, we propose that the lack of variability of *C*. *vellerosus* disjunct population is possibly due to a modern translocation of individuals by human activity, should be interpreted as an evidence that supports the hypothesis that the species recently arrived to the coast by means of long distance colonization, and not by means of a contiguous range expansion.

## Materials and methods

### Specimens analysed and sampling locations

This study was carried out in strict accordance with the recommendations for care and use of animals of the Ethical Framework of Reference for Biomedical Research, Annex II: Ethical principles for research in animals from laboratory, farms and obtained from nature, Resolution n° 1047/05 of the Ministry of Education Science and Technology, Secretariat of Science, Technology and Productive Innovation, National Council of Scientific and Technical Research (CONICET) of Argentina. Fieldwork authorization L1-00235-11 was extended by the Secretariat of Environment and Sustainable Development, Directorate of Conservation and Protected Areas after review and approval of the capture and tissue collection methods used in this study. One hundred and three specimens were collected on the field, including dry remains and run over animals in different states of conservation. Alive animals were caught by hand. Due to our own previous experience using anesthetics to take blood samples from armadillos on the field [5], in order to minimize the handling time of the animals and avoid recovery time we decided to take a small skin sample of the rim of the ear using an ear-notcher. Tissue was fixed in ethanol and the wound immediately disinfected with iodopovidone. Individuals were then released at the point of capture. Two additional samples of dried remains of putative *C*. *nationi* from Oruro province (Bolivia) were gently donated by José Carlos Pérez-Zubieta, Universidad Mayor de San Simón (Cochabamba, Bolivia). Other 35 samples (24 *C*. *vellerosus* and 11 putative *C*. *nationi*) were obtained from museum old specimens and faculty collections. Tissues and other data associated with each individual were referenced directly to each voucher specimen and stored along with a field catalog number in the collection of the IDEA (Instituto de Diversidad y Ecología Animal, CONICET-Universidad Nacional de Córdoba, Argentina). Besides, we added 10 sequences of putative *C*. *nationi* from different localities of the department of Oruro, Bolivia, available in GenBank [7] to the analysis of our sequences. Since all sequences from Bolivia belonged to the same haplotype (including the two sequences obtained in this study and those from GenBank), we collapse all Bolivian localities in one (Oruro) to facilitate configuration and visualization of H1 distribution in the Network of Fig 1 (details of the distribution are presented in S2 Table).

### DNA extraction, amplification and sequencing

DNA was extracted using the sodium dodecyl sulfate-proteinase K/phenol/RNAse method [52] and concentrated by ethanol precipitation. Dried skin samples from collections were previously subjected to a treatment to improve DNA extraction, following Moraes Barros & Morgante [53]. Partial sequences of mtDNA Control Region (CR) were amplified using the universal primers Thr-L15926 (5´-CAATTCCCCGGTCTTGTAAACC-3´), located in the neighboring tRNA-pro gene and DL-H16340 (5´-CCTGAAGTAGGAACCAGATG-3´) [54]. Amplification of double-stranded product was performed following https://www.protocols.io/view/pcr-partial-control-region-chaetophractus-456-bp-kmncu5e in a Biometra T Personal thermocycler.

Double-stranded PCR products were purified and concentrated by ethanol precipitation, examined on 1% agarose gels and directly sequenced using the same primers used for amplifications at Macrogen (Seoul, Korea). Sequences were edited with BioEdit 7.0.5.3 [55] and aligned using CLUSTAL W [56].

### Data analysis

Arlequin 3.5 [57] was used to calculate Fu’s and Tajima’s neutrality tests, nucleotide diversity, mismatch distribution, the goodness-of-fit test between the observed mismatch distribution and that expected under a expansion model using the sum of squared deviations [58, 59] and Harpending´s raggedness test [60]. We calculated a median-joining (MJ) network [61], relative frequencies among haplotypes and drawed mismatch distributions with Network 4 (www.fluxus-engineering.com). Loops were resolved following Crandall and Templeton [62].

Due to the disjunction in the species distribution and the long geographic distance that separates the Bolivian samples from the rest of specimens, we created three different dubsets in order to better understand results. They were: *Data set 1* (includes all samples), *Data set 2* (excludes samples of coastal disjunct population) and *Data set 3* (excludes samples of coastal disjunct population and samples from Bolivia). Pairwise differences, Tajima´s and Fu´s tests, mismatch distributions and the goodness-of-fit tests between observed and expected values under an expansion model were calculated for each.

## Supporting information

S1 Table Detailed information of samples. Geographic location and repository.
(XLSX)

S2 Table Individuals from Bolivian localities. Geographic information, haplotype composition and GenBank accession number.
(XLSX)
